# Understanding the human dimensions of a sustainable energy transition

**DOI:** 10.3389/fpsyg.2015.00805

**Published:** 2015-06-17

**Authors:** Linda Steg, Goda Perlaviciute, Ellen van der Werff

**Affiliations:** Department of Psychology, Faculty of Behavioural and Social Sciences, University of Groningen, Groningen, Netherlands

**Keywords:** sustainable energy transition, behavior, interventions, values, acceptability

## Abstract

Global climate change threatens the health, economic prospects, and basic food and water sources of people. A wide range of changes in household energy behavior is needed to realize a sustainable energy transition. We propose a general framework to understand and encourage sustainable energy behaviors, comprising four key issues. First, we need to identify which behaviors need to be changed. A sustainable energy transition involves changes in a wide range of energy behaviors, including the adoption of sustainable energy sources and energy-efficient technology, investments in energy efficiency measures in buildings, and changes in direct and indirect energy use behavior. Second, we need to understand which factors underlie these different types of sustainable energy behaviors. We discuss three main factors that influence sustainable energy behaviors: knowledge, motivations, and contextual factors. Third, we need to test the effects of interventions aimed to promote sustainable energy behaviors. Interventions can be aimed at changing the actual costs and benefits of behavior, or at changing people’s perceptions and evaluations of different costs and benefits of behavioral options. Fourth, it is important to understand which factors affect the acceptability of energy policies and energy systems changes. We discuss important findings from psychological studies on these four topics, and propose a research agenda to further explore these topics. We emphasize the need of an integrated approach in studying the human dimensions of a sustainable energy transition that increases our understanding of which general factors affect a wide range of energy behaviors as well as the acceptability of different energy policies and energy system changes.

## Introduction

Global climate change poses a major threat to the health, economic prospects, and basic food and water sources of billions of people (IPCC, 2014). Negative effects of global climate change are already occurring, such as extreme weather events and reductions in global food supply (IPCC, 2014). Future effects will be even more severe. Global climate change is caused by the emission of greenhouse gasses, which have steadily increased by about 1.5 times between 1990 and 2008 (Boden et al., 2012). Emissions are likely to further increase due to an increasing world population and economic growth (Kharas, 2010; Gerland et al., 2014). CO_2_ is the most important greenhouse gas, responsible for about 84% of the total emissions of greenhouse gasses (EPA, 2004). After remaining at stable levels for the past 1000 years at about 280 ppm, atmospheric concentrations of CO_2_ are now above 400 ppm (e.g., Mauna Loa Observatory, 2015). Global climate change and environmental decline are largely caused by human behavior and can thus be altered when people more consistently engage in sustainable energy behavior (Dietz et al., 2009; Pawlik and Steg, 2013; IPCC, 2014). The main human activity that emits CO_2_ is the combustion of fossil fuels for energy and transportation. For example, households are responsible for 26% of direct energy consumption in Europe by using electricity and gas for, among others, space heating, water heating, and the use of appliances (Eurostat, 2014). This figure is even higher when we also consider energy use for private transportation (32% of all energy is consumed for transportation) and embodied energy use, that is, the energy needed to produce, transport, and dispose of goods and services that households consume. For this reason, we focus on household energy behavior in this paper.

Given the urgency of combating anthropogenic climate change, and the fundamental changes needed to realize a sustainable energy transition, substantial modification of a wide range of household energy behavior is needed. These include the adoption of sustainable energy sources and technologies, the adoption of energy efficiency measures in buildings, the adoption of energy-efficient appliances, and changing user behavior to reduce total energy demand and to match energy demand to available supply of (renewable) energy carriers. Achieving these wide-scale changes in behavior requires a prominent role of social scientists in understanding how to motivate and enable people to actively contribute to a sustainable energy transition (ISSC and UNESCO, 2013; Hackmann et al., 2014; Sovacool, 2014; Weaver et al., 2014). Social scientists can study which factors cause sustainable and unsustainable energy behavior, and examine how these factors can be addressed in energy policy and energy system changes (see also Dietz et al., 2013). Besides, social scientists can study which factors determine the effectiveness and acceptability of energy system changes and policies aimed at promoting a sustainable energy transition (see also Stern, 2014).

In this paper, we review the contribution of social and environmental psychology in understanding and promoting sustainable energy behavior by individuals and households. We propose a general framework, comprising four key issues:

(1) identification and measurement of energy behaviors to be changed,
(2) examination of the main factors underlying energy behavior, including the adoption of sustainable energy resources and energy-efficient technology, investments in energy efficiency measures in buildings, and user behavior,
(3) designing and testing interventions to change energy behavior as to reduce CO_2_ emissions by households, including information, financial incentives, regulations and technological changes,
(4) studying factors underlying public acceptability of interventions and changes in energy systems.

We discuss key findings from psychological studies on these four topics, and propose a research agenda for further exploration of these topics. In doing so, we will demonstrate that many studies follow a narrow approach, by studying specific antecedents of single energy behaviors or effects and acceptability of specific policies. We emphasize the need of an integrated approach in studying the human dimensions of a sustainable energy problems that increases our understanding of which general factors affect a wide range of energy behaviors as well as the acceptability of different energy policies and energy system changes. We elaborate on this issue in the Discussion section.

## Which Behavior Changes are Needed to Promote a Sustainable Energy Transition?

A sustainable energy transition implies that future energy systems will more strongly rely on renewable energy sources, such as solar or wind energy. Hence, to realize a sustainable energy transition, we need to understand to what extent and under which conditions individuals are willing to accept and adopt renewable energy sources. Besides, to enhance the efficiency of sustainable energy systems and to meet energy demands of individuals and households across the world, total energy demand needs to be reduced, at least in developed countries. For this purpose, individuals can invest in energy efficiency, such as refurbishment of houses and adoption of energy-efficient appliances. Also, they can change their daily energy behaviors, such as reducing thermostat settings or showering time. In addition, people could refrain from certain actions to reduce energy demand (Huber, 2000). Moreover, given that the production of energy from renewable resources may strongly vary with weather conditions, renewables are not always readily available. Hence, individuals may need to balance their energy demand to the available supply of energy produced from renewable resources. Balancing energy demand and supply can be realized by shifting energy use in time, either autonomously or by installing technologies that automatically switch on or off specific appliances on the basis of the available energy supply. In addition, people could adopt storage technologies such as batteries and electric cars.

From a practical point of view, studies ideally focus on behaviors that have an important impact on total energy use and CO_2_ emissions, such as the adoption of renewable energy sources, home insulation, and space heating (Abrahamse et al., 2007; Dietz et al., 2009). Households use energy not only in a direct way, for example by using gas or electricity for cooking and heating, but also in an indirect way (Vringer and Blok, 1995; Kok et al., 2006). Indirect energy use refers to the energy requirement of the production, transportation and disposal of goods and services used by households. In European countries, about half of total household energy use reflects direct energy use, while the other half is related to indirect energy use (Kok et al., 2003; Reinders et al., 2003). Yet, only few studies examined factors underlying behavior related to indirect energy use (Gatersleben et al., 2002; Poortinga et al., 2003; Abrahamse et al., 2007). Environmental scientists have developed various tools for assessing direct and indirect energy use, such as life-cycle analysis, and input-output analysis (e.g., Kok et al., 2006). Although the exact numbers produced by these approaches are still debated and remain a topic of research (e.g., Padgett et al., 2008; Dudley et al., 2014), such tools are useful for identifying behaviors associated with relatively high levels of indirect energy use that can help social scientists to identify high impact behaviors to be studied.

As yet, different types of energy behaviors are typically studied in isolation. For example, studies have examined the adoption of renewable energy sources such as solar or wind energy (see Perlaviciute and Steg, 2014, for a review), subscription to green power tariffs (Tabi et al., 2014), investment in specific energy efficiency technologies such as electric vehicles (Schuitema et al., 2013; Bockarjova and Steg, 2014; Klöckner, 2014; Noppers et al., 2014) or energy efficient light bulbs (Reynolds et al., 2007; Lee et al., 2013), the adoption and use of specific components of smart grids (Sintov and Schultz, 2015), including smart meters (Kaufmann et al., 2013), and specific energy behaviors such as doing your laundry (McCalley and Midden, 2002) or showering (Aronson and O’Leary, 1982–1983). An important question is how these different types of behaviors are related, and how broader lifestyle effects can be realized, including, for example, adoption of renewable energy sources and energy-efficient technologies, changes in everyday energy behavior, investments in refurbishments, and acceptability of energy policy. A key issue here is whether and under which conditions engagement in one type of sustainable energy behavior is likely to spillover to other behaviors, including other types of environmental behavior such as water use and waste handling (Truelove et al., 2014). On the one hand, some studies suggest that engaging in one type of sustainable energy behavior is likely to inhibit other sustainable energy behaviors (referred to as negative spillover, the rebound effect, the Jevons paradox, or moral licensing; Thøgersen and Ölander, 2003; York, 2012; Tiefenbeck et al., 2013). For example, people were likely to increase their energy consumption after reducing their water use (Tiefenbeck et al., 2013), and they were less likely to recycle their waste after buying organic products (Thøgersen and Ölander, 2003). Research suggests that so-called compensatory green beliefs, reflecting the extent to which individuals think that engagement in one sustainable behavior legitimates not acting sustainable in another occasion, may inhibit durable sustainable energy behavior, and hence result in negative spillover effects (Kaklamanou et al., 2015). Yet, literature suggests that such negative spillover effects may be small (Gillingham et al., 2013; Blanken et al., 2015) and generally not fully offset the efficiency gains of the initial measure (Barker et al., 2009; Frondel et al., 2012). Still, little is known about how we can prevent that sustainable energy actions lead to negative spillover or “rebound” effects.

On the other hand, several studies have found positive spillover effects (Thøgersen and Ölander, 2003; Lanzini and Thøgersen, 2014). For example, people who recycled were more likely to buy organic food and use environmentally-friendly modes of transport one and two years later (Thøgersen and Ölander, 2003). Also, an increase in green buying following an intervention promoted subsequent recycling, the use of public transport, car-pooling, printing on both sides, saving water, and switching off lights (Lanzini and Thøgersen, 2014). Research suggests that such positive spillover effects are more likely when people relate the initial sustainable energy behaviors to themselves, thereby strengthening their environmental or energy-saving self-identity (Van der Werff et al., 2013, 2014a,b). More particularly, when people realize they engaged in sustainable energy behaviors (or more generally pro-environmental behaviors), they are more likely to see themselves as a pro-environmental person, which motivates them to act in line with this identity in subsequent situations. This finding is in line with self-perception theory (Bem, 1972). The question remains, however, how durable positive spillover effects are, as long-term effects have typically not been considered. Also, only few studies have tested causality regarding spillover effects. We come back to this issue in the Discussion section.

## Factors Underlying Energy Behavior

Behavioral interventions aimed to encourage sustainable energy use will be more successful if they target important antecedents of behavior, and remove significant barriers to change. Hence, it is important to examine which factors affect the likelihood that people engage in behaviors that promote a sustainable energy transition. In this section, we discuss three key factors that may influence sustainable energy behavior: people need to be aware of the need for and possible ways to contribute to a sustainable energy transition, they should be motivated to engage in the relevant behaviors, and they need to be able to do so.

### Knowledge

In general, people are well aware of the problems related to household energy use, and are concerned about these problems (Abrahamse, 2007). Yet, knowledge on the causes and consequences of climate change, as well as on the impact of human behavior on climate change is not always accurate. For example, there is still confusion about the processes that cause global warming (e.g., Bord et al., 2000; Whitmarsh et al., 2011). Also, only about half of the people know that if today’s greenhouse content in the atmosphere would be stabilized, the climate would still warm for at least another 100 years (Tobler et al., 2012). Climate change knowledge is higher among those with a higher level of education, although correlations were not strong (Tobler et al., 2012).

People have a limited understanding of the extent to which their behavior contributes to climate change. For example, only a limited number of people know that heating and cooling homes contribute to global warming (Bord et al., 2000). People have misperceptions regarding the relative contribution of different activities and processes causing global warming: generally people identify the causes of global warming more with distant activities, such as industry, than with their own actions (Whitmarsh et al., 2011).

Besides, people’s perceptions of the energy use through their own behaviors is not always accurate. This implies that they may not accurately judge which behavior changes are effective to reduce energy consumption and related CO_2_ emissions. People tend to rely on a simple heuristic when assessing the energy use of household appliances, notably the size of appliances. The larger the appliance, the more energy it is believed to use (Baird and Brier, 1981; Schuitema and Steg, 2005), which is not always true. This may lead to underestimations of the energy use of small appliances, such as chargers, and overestimations of the energy use of large appliances, such as a vacuum cleaner. In addition, people tend to underestimate the energy needed to heat water, which suggests that people are not well aware of the fact that they can save energy by showering or bathing less (Schuitema and Steg, 2005). Also, people think that higher energy savings can be realized via curtailment behaviors, such as turning off lights, than efficiency improvements, such as installing more efficient light bulbs and appliances (Attari et al., 2010), while the opposite is true according to experts. Assessing indirect energy use is even more complicated, as, typically, no information of the “embedded” energy use of products and services is provided. Indeed, people know relatively little about the energy use associated with the production, transportation, and disposal of products (Tobler et al., 2011). For example, they overestimate the environmental benefits of organic production, as well as the environmental harm of packaging and conservation of vegetables. Moreover, when assessing the environmental impact of vegetables, people mainly consider the transportation distance rather than transportation mode (Tobler et al., 2011). Also, many people do not know that meat consumption contributes to global warming through indirect energy use (Whitmarsh et al., 2011).

People may also hold misperceptions about characteristics of different types of energy sources and their effects on the environment. For example, some individuals categorized natural gas as a renewable energy source, most likely due to the connotation of the English word “natural,” while only about 55% recognized that biomass is a renewable energy source (Devine-Wright, 2003). Also, people associated bioenergy with fossil fuels due to the involved process of burning materials (Butler et al., 2013). People hold misperceptions about carbon capture and storage technology as well. For example, they associate storing CO_2_ with blowing a balloon and hence mistakenly picture CO_2_ reservoirs as big underground caverns filled with pure CO_2_ (Wallquist et al., 2010).

Knowledge about environmental and climate change problems is related to more concern about these problems, and more positive attitudes toward environmental protections (O’Connor et al., 1999). For example, people who are more knowledgeable about climate change and the causes of climate change are generally more concerned about climate change (Sunblad et al., 2009; Tobler et al., 2012; Guy et al., 2014). People with direct experiences of consequences related to climate change are more concerned about problems related to climate change, and more willing to reduce their energy use (Spence et al., 2011; Akerlof et al., 2013; Rudman et al., 2013). Individuals with right-of-centre political views and those who emphasize individual autonomy rather than collective ties are more likely to reject mainstream climate science knowledge (Kahan et al., 2010; Costa and Kahn, 2013). This is particularly likely when solutions to climate change conflict with one’s political ideology, suggesting that rejecting climate change knowledge could be a motivational phenomenon (Campbell and Kay, 2014; see also McCright and Dunlap, 2013). A correlational study in the US revealed that higher levels of science literacy and technical reasoning capacity were not related to increased concern with climate change, suggesting that lack of understanding of the science behind climate change is not the main reason for not taking climate change seriously (Kahan et al., 2012). If anything, science literacy and numeracy led to more polarized attitudes toward climate change that align with people’s worldviews, notably hierarchical, individualistic versus egalitarian, communitarian worldviews that are associated with, respectively, relatively low versus high concern with climate change (Kahan et al., 2012; cf. McCright and Dunlap, 2011a,b). The media and public and political debate (e.g., initiated by interest groups) present arguments that nourish views of climate change supporters as well as climate change deniers. In the USA, campaigns have been pursued that deny the seriousness of anthropogenic climate change, primarily emphasizing that scientists disagree about climate change, thereby aiming to create confusion among the public (McCright et al., 2013). The more individuals think that scientists disagree about climate change, the less they believe in global warming and the less they support policies to combat climate change. People with right-wing and conservative political views are more prone to doubt scientific consensus on climate change, whereas people with left-wing and liberal political views and those who participate in environmental movements are more likely to believe that scientists agree on this topic (McCright et al., 2013). At the same time, it was found that specific climate change knowledge attenuates the negative relationship between individualistic ideologies and beliefs about the existence of climate change (Guy et al., 2014).

Knowledge can affect the evaluation of pros and cons of energy alternatives. For example, the more factual knowledge respondents had about hydrogen, the more they perceived it as environmentally-friendly, but also, although to a lesser extent, as unsafe (Molin, 2005). Knowledge is not strongly related to environmental behavior, including energy behavior. Although some studies showed that more environmental knowledge increases the likelihood of pro-environmental and sustainable energy behavior somewhat (Hines et al., 1986/1987; Frick et al., 2004), other studies showed that more knowledge does not encourage pro-environmental and sustainable energy behavior (Schahn and Holzer, 1990; Kollmuss and Agyeman, 2002; Meinhold and Malkus, 2005; Vicente-Molina et al., 2013). Research suggests that different types of knowledge can predict environmental behavior differently. More specifically, only action-related knowledge (i.e., knowing what can be done about environmental problems) and effectiveness knowledge (i.e., knowing about the benefits or effectiveness of pro-environmental actions) predicted environmental behavior, although this was the case in just two out of five sub-samples included in this particular study (Frick et al., 2004). System knowledge (i.e., understanding the natural states of ecosystems and the processes within them) only affected environmental behavior indirectly, via the other two types of knowledge. These findings suggest that although knowledge may be a precondition for pro-environmental and sustainable energy behavior, it is not sufficient to promote such behavior. Notably, knowledge will have limited effects when people are not motivated to engage in sustainable energy behavior, or when they do not feel able to engage in such behaviors. We elaborate on these two factors below.

### Motivations

Whether or not people engage in sustainable energy behavior will depend on their motivation to do so. People will be more motivated to engage in sustainable energy behaviors when they evaluate the consequences of such behaviors more favorably, that is, when the behavior has relatively more benefits and less costs. Individuals can base their decisions on the evaluation of individual as well collective consequences of behavior, as we illustrate below. Next, we discuss general motivational factors, notably values, which affect how people evaluate various costs and benefits of specific sustainable energy behaviors.

People are more likely to engage in sustainable energy behavior when they believe such behavior has relative low individual costs and high individual benefits, resulting in overall positive evaluations of the relevant actions. This was found for both direct and indirect energy use. For example, people were more likely to travel by car (Bamberg and Schmidt, 2003), to purchase energy-saving light bulbs, and to consume meat when they evaluated these behaviors more favorably (Harland et al., 1999). Besides instrumental costs and benefits such as prices, time, and comfort, people may also consider affective and social costs and benefits. For example, people are more likely to engage in sustainable energy behaviors when they expect to derive pleasure from such behavior (Smith et al., 1994; Pelletier et al., 1998; Steg, 2005; Carrus et al., 2008; Gatersleben and Steg, 2012), and when they expect that others would approve of it (Harland et al., 1999; Nolan et al., 2008), and when receive information on the sustainable energy behaviours of others (Allcott, 2011). They may also engage in sustainable energy behavior because they expect that the particular behavior enhances their status, particularly when the behavior is somewhat costly, as in this case the behavior signals to others that they have sufficient resources to make altruistic sacrifices (Griskevicius et al., 2010). Similarly, the likelihood of adoption of sustainable innovations such as an electric car and renewable energy systems appeared to be higher when consumers evaluated their symbolic aspects, that is, the extent to which these innovations signal something positive about the owner or user to others and themselves, more favorably (Noppers et al., 2014). Positive symbolic outcomes may thus encourage people to adopt sustainable innovations, even though they still have some instrumental drawbacks, which is often the case in the early introduction phases. In fact, it appears that evaluations of the symbolic aspects of sustainable energy innovations more strongly predict interest in such innovations when people think the innovations have some instrumental drawbacks, probably because these drawbacks increase the signaling function on the relevant behavior (Noppers et al., 2014). Behavior has a larger signaling value for prestige and identity effects when it is somewhat costly. For example, when sustainable energy behavior is very easy, convenient or profitable, it is hard to claim that you engaged in the behavior because you care for others and the environment. Engaging in sustainable energy behavior that is somewhat costly or effortful is more likely to signal that you care about others and the environment (cf. Gneezy et al., 2012).

Some sustainable energy behaviors have clear individual benefits. For example, some people may enjoy cycling more than driving a car, saving energy at home will save money, and driving an electric vehicle may enhance people’s status, as described above. However, sustainable energy behaviors oftentimes are somewhat costly, effortful, and less pleasurable. For example, insulating your home or installing solar panels on your roof is a hassle and costs time and effort, investing in energy efficient technology can be costly, switching off appliances may be more effortful than leaving them on standby, and using particular appliances only when sufficient renewable energy sources are available limits freedom of choice. Yet, many people do engage in such behaviors, even though they are somewhat costly or effortful. What motivates people to engage in costly or effortful sustainable energy behavior?

People not only consider individual consequences of behavior, but also collective consequences. Sustainable energy behaviors benefit the environment as they result in a reduction of CO_2_ emissions (Steg et al., 2014b). People are motivated to see themselves as morally right, which may encourage sustainable energy behaviors, as this indicates that one is doing the right thing (Bolderdijk et al., 2013b). This implies that sustainable energy behavior not only results from individual considerations, but also from moral considerations. Indeed, several studies revealed that moral considerations affect sustainable energy behavior, such as the purchase of energy-saving light bulbs and meat consumption (Harland et al., 2007), electricity saving at work (Zhang et al., 2013), energy saving behaviors at home (Van der Werff and Steg, 2015), and the acceptability of energy policies (Steg et al., 2005; Steg and De Groot, 2010). Interestingly, engaging in sustainable energy behavior may make people feel good because they derive pleasure and satisfaction from doing the right thing (Bolderdijk et al., 2013b; Venhoeven et al., 2013; Taufik et al., 2014). People may even physically feel warmer by engaging in sustainable energy behavior; this phenomenon is known as a warm-glow effect (Taufik et al., 2014).

Engaging in sustainable energy behavior is likely to strengthen the environmental self-identity, that is, the extent to which a person sees himself or herself as a pro-environmental person (Cornelissen et al., 2008; Van der Werff et al., 2013, 2014a). Environmental self-identity is particularly strengthened when people engaged in pro-environmental behaviors that are somewhat costly or uncommon, probably because such behaviors are more likely to signal how pro-environmental a person is (Van der Werff et al., 2014a). As indicated above, a strong environmental self-identity is likely to encourage positive spillover effects. This implies that people may engage in a wide range of sustainable energy behavior when they realize they engaged in sustainable energy behaviors that are somewhat (but not too) costly or effortful (Van der Werff et al., 2014a).

An important question is to what extent people consider and weigh individual and collective considerations of sustainable energy behavior, and which factors enhance the likelihood that they will consider individual and collective consequences in the choices they make. Values appear to be an important factor in this respect. Values reflect life goals or ideals that define what is important to people and what consequences they strive for in their lives in general (Rokeach, 1973; Schwartz, 1992). Values are general motivational factors that can affect a wide range of evaluations, beliefs, and actions (Steg et al., 2014b). Four types of values have been found to be relevant for people’s evaluations and behavior related to sustainable energy use: hedonic values that make people focus on pleasure and comfort, egoistic values that make people focus on safeguarding and promoting one’s personal resources (i.e., money, status), altruistic values that make people focus on the well-being of other people and society, and biospheric values that make people focus on consequences for nature and the environment (De Groot and Steg, 2008; Steg and De Groot, 2012; Steg et al., 2014b).

Values affect how important people find different consequences of sustainable energy behaviors, and how they evaluate these consequences. More specially, people focus particularly on the characteristics of sustainable energy behaviors that have positive or negative implications for their important values (Steg et al., 2014b). In addition, people are more aware of environmental problems caused by their behavior when they more strongly endorse biospheric values, or less strongly endorse egoistic values (Stern et al., 1995; Nordlund and Garvill, 2002; Schultz et al., 2005; Steg et al., 2005). This in turn influences their beliefs and choices. As explained before, many sustainable energy behaviors have positive collective consequences, and negative individual consequences. In line with this, research revealed that in general, people have more favorable evaluations of and are more likely to engage in sustainable energy behaviors if they have strong biospheric and, to a lesser extent, altruistic values, while they are less likely do so if they have strong egoistic and/or hedonic values (see Steg and De Groot, 2012, for a review). Yet, in some cases strong altruistic values can inhibit sustainable energy behavior, for example, when such behavior is believed to have negative consequences for the wellbeing of others (De Groot and Steg, 2008). Strong biospheric values also affect sustainable energy behavior via one’s environmental self-identity (Whitmarsh and O’Neill, 2010; Gatersleben et al., 2012; Van der Werff et al., 2013, 2014b), in turn increasing the likelihood of positive spillover effects, as explained earlier.

### Contextual Factors

In general, people care about the environment, and endorse biospheric values. Yet, many people do not consistently engage in sustainable energy behavior. How can we explain this value-behavior gap? Besides a lack of knowledge on the environmental implications of one’s behavior, and lack of motivation to do so, sustainable energy behavior can be inhibited by various contextual factors. These contextual factors define the costs and benefits of different energy behaviors thereby influencing individual motivations (Ölander and Thøgersen, 1995; Stern, 1999; Thøgersen, 2005; Lindenberg and Steg, 2007; Steg and Vlek, 2009). For example, cycling rather than driving will be more effortful when people have to travel long distances, while subsidy schemes can make investments in solar panels or investments in energy efficient technology more affordable, which may result in more favorable evaluations of these technologies. Hence, in some cases, contextual factors facilitate sustainable energy behavior, and support individuals’ biospheric values and moral considerations. For example, the provision of recycling schemes and recycling facilities promote recycling (Guagnano et al., 1995). Interestingly, this study also showed that moral considerations were less predictive of behavior when contextual factors strongly supported the behavior (i.e., when recycling bins were provided), suggesting that when behavioral costs are very low, everyone engages in the behavior, irrespective of the strength of their biospheric values and moral considerations. In other cases, contextual factors can inhibit people to act upon their biospheric values and moral considerations (Harland et al., 1999; Diekmann and Preisendörfer, 2003; Abrahamse and Steg, 2009, 2011; Steg et al., 2011). Contextual factors even may make some behaviors simply impossible (e.g., Guagnano et al., 1995; Corraliza and Berenguer, 2000).

Besides defining the costs and benefits of sustainable energy behaviors, contextual factors can serve as cues that activate specific values in a particular situation, making it more likely that these values steer decision making in that situation (Steg et al., 2014a; Steg, 2015). For example, bikini models or chocolate can activate hedonic values; status symbols or signs of money can activate egoistic values; while Bibles, churches, statues of Justitia and environmental symbols can activate altruistic and biospheric values (Verplanken and Holland, 2002; Lindenberg and Steg, 2007; Lindenberg, 2012; Perlaviciute, 2014). Also, high behavioral costs are likely to activate values related to these costs, notably hedonic and egoistic values, which makes it less likely that people act upon their biospheric values (Steg et al., 2014a; Steg, 2015). Furthermore, signs of immoral or norm violating behavior by others can activate hedonic and egoistic values, making altruistic and biospheric values less influential in the particular choice situation. The opposite is true for cues that clearly signal that others respect norms and acted morally right (Steg et al., 2014a; Steg, 2015).

## Interventions to Promote a Sustainable Energy Transition

Various studies have examined which interventions are effective to promote a sustainable energy transition. From the 1970s, these studies focused on reducing energy demand by encouraging household energy conservation behavior and investments in energy efficiency, as to prevent the exhaustion of fossil energy sources. From the 1990s, studies focused on reducing CO_2_ emissions. Whereas initially many studies focused on encouraging energy saving behavior, recently more studies focused on promoting the adoption of energy saving technologies and ways to motivate households to balance their energy demand to the available supply of (renewable) energy. Below, we review the literature on interventions to encourage sustainable energy behavior. We first discuss structural strategies that aim to enhance people’s ability and motivation to engage in sustainable energy actions, by making such actions relatively more attractive via incentives. Second, we discuss psychological strategies that aim to increase people’s ability and motivation to engage in energy saving actions without actually changing the costs and benefits of these actions.

### Structural Strategies

As indicated earlier, some sustainable energy behaviors involve some degree of effort, discomfort or are financially costly. For example, putting on a sweater instead of turning on the heater or taking shorter showers can be perceived as less comfortable, and investing in home insulation involves initial financial investments. This implies that sustainable energy behaviors are oftentimes not pleasurable or rewarding (at least in the short term) as such. It is often assumed that people are not motivated to act sustainably unless some personal benefits are involved (Penn, 2003). This implies that external incentives would be needed to motivate people to engage in sustainable energy behavior, such as subsidies, or special arrangements such as free parking spaces for electric cars (cf. Bolderdijk and Steg, 2015). Alternatively, external incentives could make unsustainable energy use more costly or less pleasurable, for example, by introducing taxes or laws and regulations; a key issue here is that such strategies often lack public support. Incentives that are aimed at changing contextual factors that define the costs and benefits of sustainable energy choices are sometimes necessary to facilitate sustainable energy choices (Geller, 2002; Steg and Vlek, 2009; Bolderdijk et al., 2012). For example, only few people would be willing to purchase an energy efficient appliance that is more than twice as expensive as other options. Yet, perceptions of costs and benefits of behavior are not always accurate. In such cases, it may be sufficient to change the perceptions of costs and benefits of options via information strategies that aim to correct such misperceptions (Steg and Vlek, 2009; Abrahamse and Matthies, 2012).

Strategies that mainly deliver and stress incentives may be less effective than sometimes assumed, and can sometimes even be counter-effective (e.g., Asenio and Delmas, 2015; see for a review, Bolderdijk and Steg, 2015). The effects of incentives strongly depend on non-financial factors, such as ease of participating or program marketing (Carrico et al., 2011). Incentives provide a fickle basis for consistent sustainable energy choices when employed in isolation. They make people focus on immediate personal costs and benefits of behavior (Steg et al., 2014a; Steg, 2015). Consequently, people will particularly engage in sustainable energy behaviors when such behavior is extrinsically rewarding (De Groot and Steg, 2009). Indeed, it was found that positive effects of financial incentives to promote eco-driving disappeared as soon as the incentives were removed (Bolderdijk et al., 2011). In addition, external incentives can inhibit positive spillover effects when subsequent actions have no clear external reward, which is not uncommon in the energy domain (Thøgersen, 2013). For example, people who focused on economic rather than environmental reasons for one pro-environmental act, in this case car-sharing, appeared to be less inclined to engage in another sustainable behavior on a following occasion, in this case recycling (Evans et al., 2013). Similarly, if people engage in sustainable energy behavior due to rules or regulations, rather than due to autonomous choice, the behavior may have a weaker signaling value for prestige or identity effects, and therefore be less likely to strengthen environmental self-identity and to promote positive spillover. This implies that many different incentives need to be implemented to encourage wide-scale behavior changes needed to realize a successful sustainable energy transition, each increasing the relative attractiveness of the specific behavior targeted. This is overall not efficient and cost-effective. In addition, external incentives will only result in behavior changes when such changes are perceived to be worth the effort (Bolderdijk and Steg, 2015). For example, appeals emphasizing the financial benefits of tire pressure checks, which are modest, were not effective at all (Bolderdijk et al., 2013b). Many single sustainable energy behaviors yield small benefits and are therefore perceived as not worth the effort (Dogan et al., 2014). For example, unplugging a single coffee machine or microwave would save less than 6 Euros a year. Hence, although targeting extrinsic motivations by introducing incentives may be needed to promote some sustainable energy behaviors, incentives are not likely to encourage people to engage in the many sustainable energy behaviors needed in a truly sustainable energy transition.

### Psychological Strategies

For this reason, it is also important to employ strategies that target or enhance motivation to engage in sustainable energy behavior. Particularly strategies that target and strengthen individuals’ intrinsic motivation to engage in sustainable energy behavior may be promising in this respect, as such strategies are more likely to result in durable behavior changes.

To start with, information can be provided as to change consumers’ beliefs about and to increase their awareness of environmental and social problems caused by their behavior, which may enable and motivate them to help reduce these problems by changing their behavior. Research suggests that providing general information about energy problems and energy conservation indeed often leads to an increase in knowledge and awareness (Staats et al., 1996; Bradley et al., 1999), but this increase in knowledge does not necessarily translate into behavior changes (Geller, 1981; Staats et al., 1996; Gardner and Stern, 2002; Abrahamse et al., 2005). Information is more likely to encourage sustainable behavior when it resonates with people’s central values. For example, whereas an environmental campaign increased knowledge among all exposed to the campaign, it only affected sustainable behavioural intentions and policy preferences for those who strongly endorsed biospheric values (Bolderdijk et al., 2013a). More generally, information strategies have been more successful when they are tailored to the needs, wants and perceived barriers of the target population (Abrahamse et al., 2005, 2007; Thøgersen, 2005). Besides, the effects of information provision depends on the sources of the information and how people evaluate those sources (Clayton et al., in press); information is more likely to change beliefs and behavior if people evaluate the source favorably and trust the source.

People can also learn about which personal actions are effective to promote a sustainable energy transition by providing them with feedback about their energy use or energy savings that they have realized. Feedback appears to be an effective strategy for reducing household energy use (e.g., Seligman and Darley, 1977; see Abrahamse et al., 2005, for a review), although some exceptions exist (e.g., Katzev et al., 1980–1981; see Fischer, 2008). Feedback is more effective when it is given immediately after the behavior occurs, as this enhances people’s understanding of the relationship between the feedback and their behavior (Geller, 2002). Also, research suggests that the more frequently the feedback is given, the more effective it is. Positive effects have for instance been found for continuous feedback (e.g., McClelland and Cook, 1979–1980). Smart meters offer possibilities for providing immediate and frequent feedback on household energy use via different means such as websites, mobile phones, and home displays (Sintov and Schultz, 2015). Smart meters, however, typically give feedback on overall energy use, which might still tell little to people about how they can reduce their energy use. In this respect, feedback on a more detailed level, for example, on an appliance level, may be more effective (Fischer, 2008). When consumers lack the motivation or resources to consciously process information or feedback on their energy behaviors, ambient persuasive technologies can be offered that promote behavior change without the need for user’s conscious attention and hence with little cognitive effort (Midden and Ham, 2012). For example processing interactive lighting feedback, such as a light that turns green, is less cognitively demanding than processing factual feedback, such as statistics on your energy use, and may facilitate and motivate people to engage in sustainable energy behavior even in cognitively demanding situations.

Various social influence strategies can be employed to encourage sustainable energy behaviors (see Abrahamse and Steg, 2013, for a review). Social influence approaches that make use of face-to-face interaction seem most effective in this respect, such as block leader approaches, and behavior modeling. In fact, block leader approaches, in which case local volunteers help inform other people in their neighborhood about a certain issue, seem to be one of the most effective social influence strategies. Block leader approaches are particularly effective when the relevant social network has more ties (Weenig and Midden, 1991). Behavior modeling entails the use of confederates or “models” who demonstrate a recommended behavior, and appears to be an effective strategy to encourage sustainable behavior too (Winett et al., 1985; Sussman and Gifford, 2013).

Other effective social influence strategies are commitments, in which case people make a promise to engage in sustainable energy behavior, and implementation intentions, in which case people not only promise to engage in sustainable energy behavior, but also indicate how and when they will do so. Importantly, both strategies appear to have long-term effects on sustainable behavior (see Abrahamse et al., 2005; Abrahamse and Steg, 2013; Lokhorst et al., 2013, for reviews). Commitments are more effective when made in public rather than private (Abrahamse et al., 2005). Although little is known about the processes through which both strategies promote behavior changes, one plausible explanation is that they strengthen personal norms. More specifically, once people committed themselves to engage in sustainable energy behavior, they are motivated to act in line with their promise, as they want to (appear to) be consistent (Abrahamse and Steg, 2013). Another strategy that makes use of individuals’ desire to be consistent is evoking cognitive dissonance between people’s reported attitudes and behavior. Such a hypocrisy strategy appears to be effective. For example, people who first reported a favorable attitude toward energy conservation, and later were made aware of their relatively high energy usage, significantly reduced their energy use (Kantola et al., 1984; see also Focella and Stone, 2013).

Social influence strategies that generally happen in a fairly anonymous way, such as descriptive norm information, social comparison feedback, and group feedback, can also encourage sustainable behavior, but seem to be less powerful than strategies that rely on face-to-face interactions (Abrahamse and Steg, 2013). The provision of descriptive norm information, that is, providing information on the behavior of others, and social comparison feedback in which case people receive feedback about one’s own performance compared with the performance of others, and providing feedback on the performance of a group can be effective in promoting sustainable energy use, although the effect size is not very strong (see Abrahamse and Steg, 2013, for a meta-analysis). Social norm information and social comparison feedback is not very effective when most (significant) others do not act sustainably. In fact, if individuals learn that most others do not engage in sustainable energy behaviors, providing feedback on the behavior of others may even be counter effective, as people are likely to follow this norm (Brandon and Lewis, 1999; Schultz et al., 2007). Another important issue to consider is that information on the behavior of others should be credible. For example, it would be unwise to communicate that most others engage in sustainable consumption while it is obvious that this is not actually the case (cf. Terwel et al., 2009).

Besides informing people about the sustainable energy behavior of others, they can also be reminded of sustainable energy behaviors they themselves already engaged in. As explained earlier, such strategies are likely to strengthen one’s environmental self-identity, particularly when one’s previous behaviors clearly signal that one acted pro-environmentally, thereby promoting subsequent sustainable energy behaviors (Van der Werff et al., 2014a). As discussed above, the latter is more likely to be the case when people are reminded of a range of sustainable energy actions they engaged in, or when they are reminded of behaviors that were somewhat costly or uncommon. This implies an interesting paradox. On the one hand, it may be beneficial to stress that many others act sustainably, as people are likely to act in line with such descriptive norms. Yet, on the other hand, it seems that stressing that only few people acted sustainably can also encourage sustainable energy choices, via a different process, as engaging in such behavior can strengthen one’s environmental identity. An important question for future research is to understand the conditions under which each of these strategies would be most effective. For example, it may depend on whether one is a potential early adopter of sustainable energy behaviors or not.

## Acceptability of Energy Policies and Changes in Energy Systems

Energy policies and energy system changes will mostly not be implemented when they lack public support. Hence, it is important to understand what factors influence public acceptability of energy policies and energy system changes. Moral considerations affect policy support: acceptability of energy policies is higher when people are highly aware of energy problems and feel morally obliged to reduce these problems (Steg et al., 2005). Furthermore, energy policies and energy system changes are evaluated as more acceptable when they do not seriously threaten people’s freedom of choice (Poortinga et al., 2003; Steg et al., 2006; Schuitema et al., 2010; Leijten et al., 2014). More generally, people evaluate energy policies and changes in energy systems as more acceptable when these policies and changes are expected to have more positive and less negative individual and/or collective consequences (Dietz et al., 2007; Shwom et al., 2010; see Schuitema and Jakobsson Bergstad, 2012, for a review). Below, we discuss two factors that affect how people perceive and evaluate various consequences of energy policies and energy system changes, namely values and trust in involved parties. In addition, public acceptability depends on how and by whom energy policies and energy systems are developed and implemented. We describe two factors that play a crucial role in this respect, namely the distribution of costs and benefits, and public engagement and participation.

### Values and Acceptability

People are more likely to accept energy policies and changes in energy systems when these policies and changes align with and support their important values. For example, stronger egoistic values were associated with more positive evaluations of nuclear energy, probably because nuclear energy is believed to have mainly positive implications for one’s egoistic vales, such as affordable and secure energy supply. In contrast, stronger egoistic values were related to less positive evaluations of renewable energy sources, which may have negative consequences for one’s egoistic values, such as being expensive and intermittent. Similarly, stronger biospheric values were related to more positive evaluations of renewable energy sources, which are generally seen as having positive implications for one’s biospheric values, such as a reduction in CO_2_ emissions. Biospheric values were related to less positive evaluations of nuclear energy, which is believed to have negative implications for one’s biospheric values, such as contamination in case of nuclear accidents (Corner et al., 2011; De Groot et al., 2013; Perlaviciute and Steg, 2015). Interestingly, people’s value-based judgements of energy sources may affect their evaluations of various consequences of these energy sources, including consequences that should not be particularly important to them given their values. For example, people with strong egoistic values were most likely to ascribe positive environmental consequences to nuclear energy, such as a reduction of CO_2_ emissions. People with strong biospheric values evaluated personal consequences of renewable energy sources more positively, such as costs and the security of energy supply, even though these consequences are probably not very important to them given their specific values (Bidwell, 2013; De Groot et al., 2013; Perlaviciute and Steg, 2015). This suggests that people base their evaluations of energy policies and changes in energy systems primarily on aspects that are most relevant for their important values, which will guide their acceptability ratings. These value-based acceptability judgements may further affect the evaluation of other characteristics of these policies and energy system changes, which may be less important to people based on their values. In other words, people are likely to evaluate energy policies and changes in energy systems in an overly positive or negative way that is in line with their value-based judgements.

Interventions aimed at strengthening public support for sustainable energy policies and energy system changes will be more effective if they target values that underlie people’s evaluations and acceptability ratings (cf. Bolderdijk et al., 2013a). Focusing merely on how people evaluate various consequences of these policies and energy system changes may be misleading, given that (some of) these evaluations can be colored by people’s values-based judgements and not reflect the actual concern people have, as we explained above. For example, people may evaluate renewable energy sources or energy efficient technology negatively, primarily because they expect negative consequences for their egoistic values related to increased costs and/or intermittency. Yet, as a consequence, they may also evaluate the environmental consequences of renewable energy sources or energy efficient technology negatively, in line with their value-based judgements. In this case, targeting the environmental consequences in intervention strategies will probably not change their acceptability ratings, as the acceptability judgements were hardly based on the evaluation of the environmental consequences in the first place. In this case, introducing subsidies for adopting renewable energy or improving the functionality of energy systems could be more motivating for them; such strategies could at the same time enhance intrinsic motivation to support durable changes in behavior, as explained above. Interestingly, while privacy concerns with regard to energy use monitoring technology such as smart metering may hinder acceptability of such technology, a study found that privacy concerns may be underpinned by the costs and benefits that people expect from such technology for them personally (Bolderdijk et al., 2013c). More specifically, privacy concerns were most prominent when people anticipated negative individual consequences (e.g., paying more for energy use) from implementing the monitoring technology. Communicating the individual benefits of such technology (e.g., the possibility to save money) alleviated privacy concerns. A thorough understanding of which values actually underlie people’s evaluations and acceptability ratings is therefore crucial for developing effective intervention and communication strategies.

### Trust in Involved Parties and Acceptability

Sustainable energy transitions entail multiple aspects, such as complex energy technology, that go far beyond the knowledge and expertise of consumers. People therefore need to rely on other parties, such as developers, governments, and scientists, to develop their views of different aspects related to sustainable energy transitions. The extent to which people trust these parties will influence acceptability of energy policies and changes in energy system (Whitfield et al., 2009; Huijts et al., 2012; Perlaviciute and Steg, 2014). Trust in involved parties will especially affect evaluations and perceptions when people have little knowledge about the proposed energy policies or energy system changes (cf. Siegrist and Cvetkovich, 2000). Trust can influence the perceived costs and benefits of sustainable energy transitions. For example, the more people trusted the parties involved in managing hydrogen systems, the more benefits and the less risks they ascribed to hydrogen as an energy carrier in cars and busses (Montijn-Dorgelo and Midden, 2008). The effects of trust on perceived risks and benefits were mediated by general attitudes toward hydrogen, in this study conceptualized as general affective evaluations (Montijn-Dorgelo and Midden, 2008).

People base their trust judgements on the perceived competence and the perceived integrity of the involved parties (Earle and Siegrist, 2006; Terwel et al., 2009). More specifically, it is not only important whether people think that the parties involved have sufficient knowledge and expertise, but also how these parties have performed in the past, whether people perceive them as open, honest, and taking their interests into account, and whether people think these parties endorse values similar to their own values (Earle and Siegrist, 2006). In general, people tend to trust universities and NGO’s more than companies and governments, although local governments are typically trusted more than national governments. This is likely to be driven by the perceived values and motivations of these actors. Specifically, people may assume that companies primarily value profit making, which, especially in the energy sector, can be seen as conflicting with public interests. In a study on sustainable energy transitions in the UK, people expressed much support for shifting toward renewable energy sources, but at the same time they expressed their concern whether the energy companies are capable of realizing sustainable energy transitions in a way that aligns with societal and environmental values (Butler et al., 2013). Lack of trust in energy companies can also elicit privacy concerns related to, for example, smart metering technology, which can weaken public support for the proposed sustainability measures (Butler et al., 2013). Interestingly, a study on acceptability of CO_2_ storage found that when people perceived themselves and professional parties as sharing similar goals and values, they expected these parties to not only have good intentions but also sufficient skills and competencies to pursue these intentions (Huijts et al., 2007). This again shows that values play an important role in public acceptability of energy policies and changes in energy systems, and that values can affect trust in involved parties.

### Distribution of Costs and Benefits

Acceptability of energy policies and energy systems changes not only depends on their benefits, costs and risks, but also on how these benefits, costs and risks are distributed among groups involved. Sustainable energy transitions will be seen as unfair if certain groups in society face most of the costs, while other groups in society mainly enjoy the benefits, which may reduce their acceptability (Schuitema and Jakobsson Bergstad, 2012). For example, communities hosting renewable energy technologies such as wind farms may experience noise and visual hinder, while the possible benefits such as reduced CO_2_ emissions, affordable energy, and energy independence are shared on a national or even global scale. As a consequence, people may oppose these technologies.

Fair distribution of costs and benefits can be pursued in multiple ways, which are not mutually exclusive. First, risks and costs of energy policies can be reduced as much as possible in order to secure public acceptability. For example, technical solutions can be sought to reduce the noise caused by wind turbines, and costs of renewable energy sources can be reduced via subsidies. A second (parallel) strategy to pursue a fair distribution of costs, risks and benefits is providing additional benefits to those exposed to most costs and risks. For example, individuals can be financially compensated, or developers of renewable energy projects could establish local funds that can be used to reduce energy bills for local people, to stimulate local economy, or to create or expand local facilities (e.g., sports facilities; Walker et al., 2014). It has been proposed that collective benefits (e.g., investing in local facilities) are less likely to be seen as “bribes” by citizens than individual financial compensations (e.g., one-time payments to residents; Ter Mors et al., 2012). However, this proposition has not been empirically tested. Interestingly, the amount of compensation may be less important for acceptability judgements than who will benefit from the compensation. For example, people prefer royalties from a wind energy project to be allocated to local funds rather than to state funds (Krueger et al., 2011). This is probably because it is seen as more fair when local communities benefit from hosting energy infrastructure than when benefits are allocated to state funds (cf. Schuitema and Steg, 2008). Yet, financial compensation to local funds will not enhance acceptability and may even backfire when such compensations are perceived as attempts to “buy local support” (Walker et al., 2014; cf. Ter Mors et al., 2012).

### Public Involvement

People may be more likely to accept energy policies and changes in energy systems if they believe that the decision-making process is fair, and if they feel they are sufficiently involved in decision-making and that their interests are considered (Huijts et al., 2012; Perlaviciute and Steg, 2014). Public involvement can take place at different levels, which will affect acceptability differently (Devine-Wright, 2011). Information provision is a necessary pre-condition for public involvement: decision-making processes need to be transparent and people should be fully informed from the beginning, rather than only afterwards when all decisions are made. Yet, information provision alone is a passive form of public involvement and is often not sufficient to secure public support for energy policies and energy system changes. Higher levels of public involvement include active public engagement in decision-making (Devine-Wright, 2011). Several case studies on renewable energy projects have concluded that technocratic top-down decision making processes inhibit public acceptability, while collaborative approaches taking community concerns into account enhance acceptability (Wolsink, 2007, 2010; Wolsink and Breukers, 2010; Walker and Devine-Wright, 2008). Public engagement means not only that people will have an opportunity to express their opinion, but also that their opinion is seriously considered in decision-making and can have an actual impact on decisions on energy policies and changes in energy systems (see Dietz and Stern (2008), for a review of dimensions and assessment criteria of participatory processes). People consider decisions more acceptable if they have been actively involved in the decision-making process (also conceptualized as legitimacy; Dietz and Stern (2008), Schuitema and Jakobsson Bergstad, 2012). Sometimes, however, people are given an opportunity to express their opinion, while their opinion is eventually not taken into account and cannot change energy policies. Such “fake” engagement can have even more negative effects on public support than no engagement at all, by diminishing trust in involved parties, as discussed above.

## Discussion

In this paper, we discussed factors influencing sustainable energy behavior by individuals and households. We proposed a general framework to study ways to understand and encourage sustainable energy behaviors needed to promote a sustainable energy transition, comprising four key issues. First, we argued that a sustainable energy transition involves changes in a wide range of energy behaviors, including the adoption of sustainable energy resources and energy-efficient technology, investments in energy efficiency measures in buildings, and changes in energy use behavior. Besides, not only direct energy use should be considered, but also indirect energy use, that it, the energy used to produce, transport and dispose of products. Second, we proposed that it is important to examine main factors underlying different types of sustainable energy behaviors. We discussed three main factors influencing such behavior that are closely intertwined: knowledge, motivations, and contextual factors. Third, it is important to test the effects of interventions aimed to promote sustainable energy behavior by changing important antecedents of these behaviors. In this respect, it is not only important to study structural strategies that affect the actual costs and benefits of behavior, but also psychological strategies that affect how people perceive and evaluate different pros and cons of behavioral options. Fourth, as policies and energy system changes will probably not be implemented when they are not supported by the public, it is important to understand which factors affect the acceptability of energy policies and energy system changes. We discussed that acceptability judgements depend on the perceived benefits, costs and risks of energy policies and energy system changes, and argued that these depend on people’s values and trust in the parties involved. Besides, perceived fairness plays a role, which depends on the distribution of benefits, costs and risks, and the level of public involvement in the decision making process.

Our review reveals that many studies followed a narrow approach, by studying specific antecedents of single energy behaviors or effects and acceptability of specific policies or energy system changes. We emphasize the need of an integrated approach in studying the human dimensions of a sustainable energy transition that increases our understanding of which general factors affect a wide range of energy behaviors as well as the acceptability of energy policies and energy system changes. Below, we propose a research agenda for studying the human dimensions of a sustainable energy transition.

### Research Agenda for Studying the Human Dimension of a Sustainable Energy Transition

Future energy systems will likely more strongly rely on renewable energy sources, such as solar or wind energy. To realize a sustainable energy transition, we need to study a range of sustainable energy behaviors in an integrated way. First, we need to understand to what extent and under which conditions individuals and households are willing to accept and adopt different renewable energy sources. Second, to enhance the efficiency of sustainable energy systems and to meet energy demands of individuals and households across the world, total energy demand needs to be reduced. For this purpose, we need to systematically study factors that increase the efficiency of energy systems. More particularly, we need to understand which factors affect investments in energy efficiency, such as refurbishment of houses and adoption of energy-efficient appliances. Also, we need to understand which factors affect daily energy use, such as thermostat settings or showering time. Third, given that the production of energy from renewable resources may strongly vary with weather conditions and that renewables are not always readily available, we need to study preferences for and acceptability of different ways to balance demand and supply of energy produced from renewable resources. Are people willing to shift energy use in time as to balance energy demand and supply, either autonomously or via automated technologies? Or do they prefer storage facilities such as “power-to-gas,” batteries and electric cars? Which factors influence the preferences for these different solutions? Fourth, besides reducing direct energy use, it is also important to consider indirect energy use which comprises about half of total household energy use. More research is needed on the extent to which people are aware of indirect energy use, and whether and under which conditions they consider indirect energy use in the decisions they make. Ideally, studies include measures of actual energy behavior and actual energy use, rather than only behavioral intentions or self-reported behavior (cf. Gatersleben et al., 2002). Various technologies have become available that enable objective measures of behavior and energy use, such as smart meters and smart plug systems.

When studying these different types of sustainable energy behaviors in an integrated way, it is important to understand factors increasing the likelihood of possible negative versus positive spillover effects. More specifically, it is important to examine the conditions under which engagement in sustainable energy behaviors gives people the feeling that they are licensed to refrain from other sustainable energy behaviors, thereby causing negative spillover. Moreover, it is important to understand how to prevent that sustainable energy behavior leads to negative spillover or “rebound” effects. Similarly, future research can examine under which conditions positive spillover effects are more likely, increasing the likelihood that people are willing to engage in many different sustainable energy behaviors over and again, which is needed to realize a truly sustainable energy transition. As discussed earlier, positive spillover effects seem more likely when people ascribe the initial behavior to themselves, thereby strengthening their environmental self-identity. Future research is needed to systematically test under which conditions the environmental self-identity will be particularly strengthened after engaging in sustainable energy behaviour, and which factors motivate people to act in line with this identity over and again (Whitmarsh and O’Neill, 2010; Truelove et al., 2014). More generally, future research can study the processes underlying positive and negative spillover effects in more depth. Self-perception theory, goal theory and cognitive dissonance theory provide possible theoretical explanations for the processes through which spillover effects occur (e.g., Thøgersen and Crompton, 2009; Thøgersen and Noblet, 2012). The question remains under which circumstances which theoretical explanation is most plausible.

Spillover effects are typically studied in lab studies focussing on one-off environmental behaviors (Truelove et al., 2014). A sustainable energy transition requires that people engage in many different sustainable energy behaviors over and again, for example when they choose whether to take a shower, what products or appliances to buy, which appliances to use in their homes, and which energy carriers to use. Future research is needed to study the scope of spillover effects, that is, which type of behaviors are particularly influenced, how many behaviors are influenced, and the pattern of spillover effects over a longer period of time. To be able to establish causality, longitudinal experimental designs need to be employed. From a broader sustainability perspective, not only spillover effects within the energy domain should be considered, but also spillover across various types of environmental behavior such as energy use, water use and waste handling.

In order to better understand why people would or would not engage in a wide range of sustainable energy behaviors, it is important to study general antecedents of such behaviors, such as values. Our review suggests that particularly strong biospheric values can create a stable and reliable basis for many sustainable energy behaviors, even if these behaviors have some personal costs. Yet, people do not always act upon their biospheric values, for example because they are not able to do so due to contextual restrictions or because cues in a given situation activate other conflicting values. It is important to study under which conditions people are more or less likely to pursue their biospheric values and how biospheric values can be activated by situational cues, so that they are more likely to steer decisions in a particular situation. Next, we know yet very little about the extent to which people’s value priorities change. Although values are considered to be relatively stable across time, changes in values have been documented due to, for example, significant life events (Steg et al., 2014a). Future research could shed more light on factors that make people reconsider the importance of their values, in particular the importance of biospheric values, for example through intensive environmental education programs.

Knowledge about energy problems and ways to reduce these problems can be an important precondition to promote a sustainable energy transition. In this respect, it is important to study factors that determine whether or not knowledge and information lead to more sustainable energy behavior. One important question is which types of knowledge are particularly important to change people’s concerns, beliefs, and perceived efficacy to engage in sustainable energy behavior, and their actual behavior. Next, given new developments in the sustainability domain, such as smart metering systems that can offer detailed feedback on one’s energy use and savings, it is important to study which type of feedback (e.g., financial, environmental, or social comparison feedback) is most effective to encourage sustainable energy behavior, and under which conditions these changes are most likely.

Sustainable energy transitions will bring changes in energy systems, and involve the implementation of different energy policies. The extent to which these policies and system changes can be implemented will depend on public acceptability. This review suggests that values and trust affect how people perceive different benefits, costs and risks of energy systems and energy policies. Future research can examine how and under which conditions values and trust particularly affect perceived consequences and acceptability of energy system changes and energy policies. Also, more systematic research is needed on factors influencing perceptions of the distribution of costs and benefits of policies and energy system changes, and on ways to enhance distributive fairness by reducing the (local) costs and risks, and enhancing the benefits of energy transitions. In this respect, it is particularly important to systematically study how different types of benefits or compensations (e.g., financial versus in-kind) and differences in how these benefits are allocated (e.g., individual versus local versus national) influence public acceptability.

Active public involvement in decision-making can foster sustainable energy transitions that are acceptable to the public. The current conceptualisation of public involvement entails many components that are potentially important for public acceptability, including transparency in information provision and decision-making, possibilities to voice public opinion, and integrating public opinion in decision-making. It is important to systematically study the effects of these different components of public involvement on public acceptability, and to study how public involvement can best be organized to enhance public support for proposed solutions by carefully taking into account the interests of different stakeholders (see also Dietz and Stern, 2008, for research priorities in this area).

A sustainable energy transition to combat anthropogenic climate change involves fundamental and wide-scale changes in human perceptions, preferences and behavior. Achieving these changes in perceptions, preferences and behavior calls for a prominent role of social scientists in understanding how to motivate and enable people to actively contribute to a sustainable energy transition. We proposed an integrated approach to address this challenge that increases our understanding of how to motivate and empower individuals and households to engage in a wide range of sustainable energy behaviors that are needed to encourage a sustainable energy transition.

### Conflict of Interest Statement

The authors declare that the research was conducted in the absence of any commercial or financial relationships that could be construed as a potential conflict of interest.
